# Evaluation of hard and soft tissue changes around dental implants with osseodentification in contrast with conventional osteotomy technique: An *in vivo* study

**DOI:** 10.6026/973206300213640

**Published:** 2025-10-31

**Authors:** Amit Siwach, Anil Sharma, Ramesh Kumar, Omar Mukhtar, Shruti Singh, Krati Singh

**Affiliations:** 1 Department of Prosthodontics and Crown & Bridge, Kalka Dental College, Meerut, Uttar Pradesh, India

**Keywords:** Bone density, dental implants, osseodensification, osteotomy, soft tissue

## Abstract

The effects of osseodensification and conventional osteotomy techniques on dental implants is of interest. The *in vivo* research focused on hard and soft tissue changes around dental implants placed in partially edentulous patients. Data shows that osseodensification improved probing depth, bleeding index and bone density, with significant differences observed at 3 and 6 months. Thus, we show that osseodensification enhances both soft tissue health and bone preservation, supporting its use in cases with compromised bone quality. Future research is needed to explore long-term outcomes and optimize protocols.

## Background:

The goal of modern dentistry is to restore patient's teeth to normal contour, function, comfort, aesthetics, speech and health [1]. With advancements in dental healthcare, dental implants have become a highly successful and predictable treatment option, significantly improving the quality of life for individuals [2]. These implants are extensively used for rehabilitating both partially and completely edentulous patients, offering stable and aesthetically pleasing outcomes. The history of dental treatments dates back thousands of years, with archeological evidence showing that humans in earlier times attempted to replace missing teeth with root-form implants [3]. In modern times, tooth replica implants were introduced as early as 1969, though these polymethyl methacrylate analogues were encapsulated by soft tissue. The first use of titanium as an implantable material occurred in 1940 by Bothe and in 1965, Branemark placed the first titanium dental implant into a human volunteer [4]. In 1969, Branemark demonstrated osseointegration, the process where titanium implants form a direct, structural and functional contact with bone, marking a milestone in implant dentistry [5]. Dental implants are surgical components that anchor prosthetic restorations by integrating with jawbone through osseointegration-bonding of materials like titanium to bone [6]. Osseointegration depends on factors such as implant material, surface, design, bone quality, surgical technique and loading conditions. Advances in implant surfaces and techniques have shortened healing times by improving primary stability (mechanical fixation) and accelerating secondary stability (bone growth) [7]. Osseodensification, introduced by Salah Huwais in 2013, uses non-cutting burs to compact and autograft bone during osteotomy, increasing density and stability, especially in low-density areas like the posterior maxilla. Unlike traditional drilling, it expands bone outward, forming a densification layer [8]. Studies show enhanced stability compared to conventional drilling or the osteotome technique (Summers, 1994), which sometimes impedes osseointegration. Assessing bone density now aided by cone beam CT guides implant planning. Adequate keratinized tissue around implants is vital to resist infection and ensure long-term success. While osseodensification shows promise, further research is needed to confirm its efficacy [9]. Therefore, it is of interest to describe the hard and soft tissue changes around dental implants placed using osseodensification, compared with the conventional osteotomy technique.

## Methodology:

This *in vivo* study was conducted at the Department of Prosthodontics and Crown & Bridge, Kalka Dental College, Meerut, Uttar Pradesh, involving partially edentulous patients (both males and females) aged between 25 and 50 years. A total of 30 dental implants were placed. All treatment options were thoroughly discussed with the patients and they were informed about the advantages and disadvantages of implant treatment. After careful consultation, the patients were motivated to proceed with the treatment. Selection for the study was based on predefined inclusion and exclusion criteria and only patients who were ready for surgical placement and subsequent follow-up were included. The study began with a diagnostic appointment, where patients were selected based on the inclusion and exclusion criteria. Comprehensive clinical examinations were conducted, followed by radiographic assessments using cone beam computed tomography (CBCT) to examine the morphologic characteristics of the proposed implant sites and the surrounding structures. Once the diagnosis was confirmed, patients were provided with detailed information.

## Exclusion criteria comprised:

History of drug or alcohol abuse, immunological diseases such as acquired immune deficiency syndrome (AIDS) or scleroderma, bruxism, temporomandibular disorders, chronic bone diseases, psychiatric disorders, uncontrolled diabetes, need for bone augmentation procedures, inability to adhere to follow-up, incapacity to give informed consent and participation in other clinical trials. The medical history of each patient was recorded, focusing on cardiac problems, kidney, urinary tract, gastrointestinal, respiratory, endocrine and nervous system disorders, abnormal bleeding tendencies, drug reactions, substance abuse and psychological issues. Dental history was also evaluated to understand the cause and duration of tooth loss, which could be due to periodontal disease, caries, trauma, or neglect. A thorough oral health assessment was conducted to evaluate the remaining teeth and treat any sources of infection, periodontal disease, or dental caries before implant therapy. Extra oral and intraoral examinations were performed, with a focus on lymphadenopathy, thyroid hypertrophy and soft tissue examination. Vital signs including blood pressure, pulse, temperature and respiration were recorded, ensuring they were within normal ranges. Blood tests were carried out for systemic diseases and surgical evaluation, including complete blood count, hemoglobin, platelet count and random blood sugar levels.

## Eligible patients who met the inclusion criteria were divided into two groups:

Group 1, where 15 implants were placed using conventional osteotomy and Group 2, where 15 implants were placed use osseo-densification drilling. Diagnostic instruments included CBCT and Color Vue plastic probes, along with basic examination tools such as mouth mirrors and periodontal probes. Surgical instruments comprised an ADIN Implant surgical kit, which included drills, ratchets, hex drivers, implant drivers, paralleling pins and other tools for implant placement. The Densah Bur kit (Versah, USA) was specifically used for osseodensification, a technique where bone tissue is compacted and auto-grafted, enhancing implant stability. The physio dispenser (X Cube) was used to control the speed and torque during implant placement, reducing the risk of bone damage. Prior to surgery, patients received a prophylactic dose of 2 gm Amoxicillin. The surgical site was sterilized using 5% betadine and the patient was asked to rinse with 0.12% chlorhexidine. Local anesthesia (Lignocaine with adrenaline 1:100000) was administered and a mid-crestal incision was made, followed by full-thickness mucoperiosteal flap elevation. Osteotomy was performed using either conventional or osseodensification techniques, followed by implant placement with the help of a torque wrench and implant driver. After placement, abutments were attached and closed tray implant-level impressions were made using PVS impression material. Finally, healing abutments were placed and postoperative instructions were provided.

Various indices were recorded for evaluating the hard and soft tissue changes, including sulcular bleeding index (SBI) and probing depth. The modified sulcus bleeding index was used to assess bleeding on probing around the implants, with scores recorded at baseline, 3rd and 6th months. Probing depth measurements were made using the Color-Vue plastic probe at the same intervals. Additionally, CBCT was used to assess crestal bone loss and bone density was measured at the mesial, distal, lingual and buccal aspects of the implant sites, with measurements taken both preoperatively and postoperatively.

## Results:

This study aimed to evaluate the clinical and radiographic outcomes of dental implants placed using conventional osteotomy and osseodensification techniques. The focus was on probing depth, bleeding index, bone density and crestal bone loss at various time points (baseline, 3 months and 6 months). Table 1 (see PDF) presents the descriptive statistics and intercomparison of probing depth between conventional osteotomy and osseodensification techniques at baseline, 3 months and 6 months. At baseline, the mean probing depth for both groups was similar (3.9 mm for both conventional and osseodensification). The intergroup comparison showed no statistically significant difference at baseline (p=0.159). However, at 3 months and 6 months, probing depths were significantly lower in the osseodensification group compared to the conventional osteotomy group (p<0.05). Specifically, the mean probing depth at 6 months was 3.37 mm in the conventional group and 3.12 mm in the osseodensification group (p=0.005). Table 2 (see PDF) shows intragroup comparisons for probing depth in the conventional osteotomy group. Statistically significant reductions in probing depth were observed at all time points (baseline to 3 months, 3 months to 6 months and baseline to 6 months) with p-values ranging from 0.001 to 0.019. Table 3 (see PDF) presents similar findings for the osseodensification group, where significant reductions in probing depth were observed between all time points, with p-values ranging from 0.007 to 0.000. Table 4 (see PDF) compares the bleeding index between conventional osteotomy and osseodensification groups at baseline, 3 months and 6 months. At baseline, the bleeding index was similar between both groups (0.76). The intergroup comparison at 3 months showed that the conventional group had a higher mean bleeding index (0.60) compared to the osseodensification group (0.53) and this difference was statistically significant (p=0.017). At 6 months, the bleeding index continued to favor the osseodensification group, with a lower score of 0.20 compared to 0.27 in the conventional group (p=0.031). Table 5 (see PDF) shows intragroup comparisons for the bleeding index in the conventional osteotomy group. Statistically significant reductions in bleeding were observed between baseline and 6 months (p=0.037), while no significant difference was noted between baseline and 3 months (p=0.886). Table 6 (see PDF) shows similar results for the osseodensification group, where the bleeding index significantly decreased from baseline to 3 months (p=0.000) and from 3 months to 6 months (p=0.041). Table 7 (see PDF) compares bone density at various sites (crestal, coronal third, middle third, apical third and apex) for both groups before implant placement. The osseodensification group showed higher bone density at the crestal site (856.40 ± 330.24) compared to conventional osteotomy (802.37 ± 305.34), but the difference was statistically non-significant (p=0.797). Significant differences were observed at the coronal third, where osseodensification had a higher mean value of 1001.00 ± 347.20 compared to conventional osteotomy (952.17 ± 392.21) with a p-value of 0.015. Table 8 (see PDF) presents post-operative bone density at 6 months. At the crestal site, osseodensification again showed a higher mean (830.00 ± 386.29) compared to conventional osteotomy (753.20 ± 341.08), with a significant p-value of 0.001. Similarly, osseodensification showed higher bone density at the coronal third (1028.87 ± 341.90) compared to conventional osteotomy (961.47 ± 327.06), with a p-value of 0.036.

## Discussion:

This study presents robust evidence supporting the clinical advantages of osseodensification over conventional osteotomy techniques in dental implant placement. Our findings show significant improvements in various clinical parameters, including probing depth, bleeding on probing, bone density and crestal bone preservation, when using the osseodensification technique. These results are consistent with and extend, previous research in the field. In terms of soft tissue outcomes, our study demonstrated that osseodensification achieved significantly lower probing depths compared to conventional osteotomy. At 3 months, osseodensification showed a mean probing depth of 3.33 ± 0.38 mm compared to 3.71 ± 0.60 mm in conventional osteotomy and this difference became more pronounced at 6 months. The findings align with those of Pai *et al.* (2018) [10], who also reported improvements in peri-implant soft tissue health with osseodensification, attributed to osseodensification bone-preserving method. By maintaining the natural bone structure and vascularity during osteotomy, osseodensification creates a more favorable environment for soft tissue attachment, potentially reducing peri-implant inflammation and enhancing long-term soft tissue stability. Our bleeding on probing results further reinforces the benefits of osseodensification for peri-implant health. While both groups showed comparable baseline bleeding on probing scores, the osseodensification group had significantly lower scores at both 3 months and 6 months, indicating reduced inflammation and improved tissue healing. This aligns with Rahimnejad *et al.* (2024 [11], who linked minimally traumatic bone preparation techniques, like osseodensification, with better soft tissue integration and reduced inflammation. The reduced inflammation in the osseodensification group could be attributed to the mechanical compaction of bone, which stabilizes the bone-implant interface and minimizes soft tissue irritation. The most compelling evidence for osseodensification superiority is found in our bone density measurements. Pre-operative bone densities were comparable between the two groups, but post-operative evaluations revealed that osseodensification resulted in higher bone density at all regions measured: crestal, coronal, middle and apical. This increase in bone density is consistent with the findings of Bergamo *et al.* (2021) [12], who also reported improved bone density with osseodensification. Osseodensification's auto grafting effect, caused by compaction of bone particles along the osteotomy walls, likely contributes to the denser bone-implant interface, which enhances secondary stability and long-term implant success. Moreover, crestal bone preservation was superior in the osseodensification group, with less bone loss at both mesial and distal sites compared to conventional osteotomy. These results are in line with Prasad *et al.* (2011) [13], who suggested that osseodensification reduces osteotomy trauma, leading to better bone retention and more favorable stress distribution. The ability to preserve crestal bone is crucial for long-term implant success, especially in aesthetic zones and cases requiring immediate loading. While the study provides strong evidence supporting the advantages of osseodensification, several limitations should be considered. The sample size of 15 implants per group, though adequate for statistical significance, may benefit from expansion in future studies. The 6-month follow-up period does not provide insight into the long-term outcomes of osseodensification. Furthermore, Omidian *et al.* (2025) [14] noted that osseodensification effectiveness is highly dependent on the operator's experience and technique sensitivity, emphasizing the need for proper training and standardized protocols to optimize clinical results.

## Conclusion:

The significant advantages of osseodensification over conventional osteotomy in dental implant placement, including improved soft tissue health, enhanced bone density and better crestal bone preservation is shown. These benefits make osseodensification a promising technique, particularly in cases with compromised bone quality. Future research should focus on long-term outcomes and standardized protocols for broader clinical application.
